# A Successful Prevention of Reintubation Using the Mechanical Insufflation-Exsufflation in a Critically Ill Patient With Impaired Airway Mucus Expectoration: A Case Report

**DOI:** 10.7759/cureus.47776

**Published:** 2023-10-27

**Authors:** Chisa Nishida, Shu Utsumi, Toshiki Sera, Shinichiro Ohshimo, Nobuaki Shime

**Affiliations:** 1 Department of Emergency and Critical Care Medicine, Hiroshima University Graduate School of Biomedical and Health Sciences, Hiroshima, JPN

**Keywords:** intensive care unit, respiratory care, musculoskeletal rehabilitation, reintubation, airway management, mechanical insufflation-exsufflation, cough augmentation, acute respiratory failure

## Abstract

We report a case of post-extubation respiratory failure due to insufficient airway mucus expectoration that was successfully treated using mechanical insufflation-exsufflation (MI-E). A 32-year-old woman with a long-term history of steroid therapy for Blau syndrome was admitted to our intensive care unit with refractory hypoxemia due to pneumonia associated with the novel coronavirus disease 2019. Mechanical ventilation with veno-venous extracorporeal membrane oxygenation (VV-ECMO) was required due to severe hypoxemia. She was weaned from VV-ECMO on the 10th day and extubated on the 13th day. A few hours after extubation, she presented respiratory distress due to massive pulmonary atelectasis caused by sputum accumulation as a result of the impaired cough reflex. MI-E was applied to facilitate coughing and sputum expectoration. MI-E dramatically improved the atelectasis and prevented reintubation. This case suggests that MI-E, which is primarily used to treat chronic neuromuscular diseases, may also be effective in treating acute respiratory failure.

## Introduction

Secretion impairment is a common cause of post-extubation respiratory failure [1]. Standard care includes respiratory physiotherapy to maintain airway clearance. Mechanical insufflation-exsufflation (MI-E) is a cough augmentation device involving rapid pressure shifts that facilitate the mobilization of peripheral sputum and stimulate natural coughing. MI-E is considered an efficient respiratory physiotherapy for patients with chronic cough dysfunction [2], including those with neuromuscular disease (NMD). Despite the increasing interest in the effectiveness of MI-E in patients with acute illness [3,4], there remains limited evidence supporting its use in intensive care unit (ICU) settings [5]. Herein, we report a case of post-extubation respiratory failure caused by insufficient airway mucus expectoration that was successfully treated with MI-E.

## Case presentation

**Figure 1:**
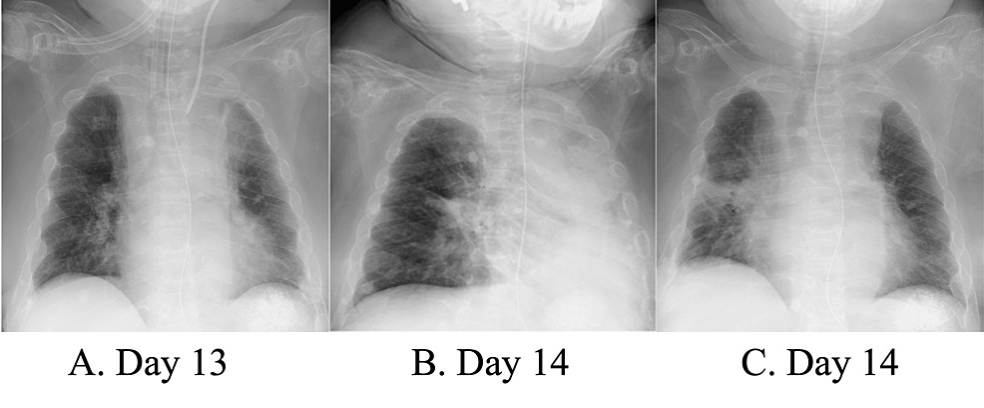
Serial changes in chest radiographs (A) A chest radiograph scan obtained on the 13th day after admission (just before extubation) showing no significant abnormal findings; (B) A chest radiograph scan obtained on the 14th day (a few hours after extubation) showing massive atelectasis in the left lung field; (C) A chest radiograph scan obtained a few hours after initiation of MI-E showing marked improvement of atelectasis in the left lung field, which was probably caused by sputum retention. MI-E: mechanical insufflation-exsufflation

**Figure 2:**
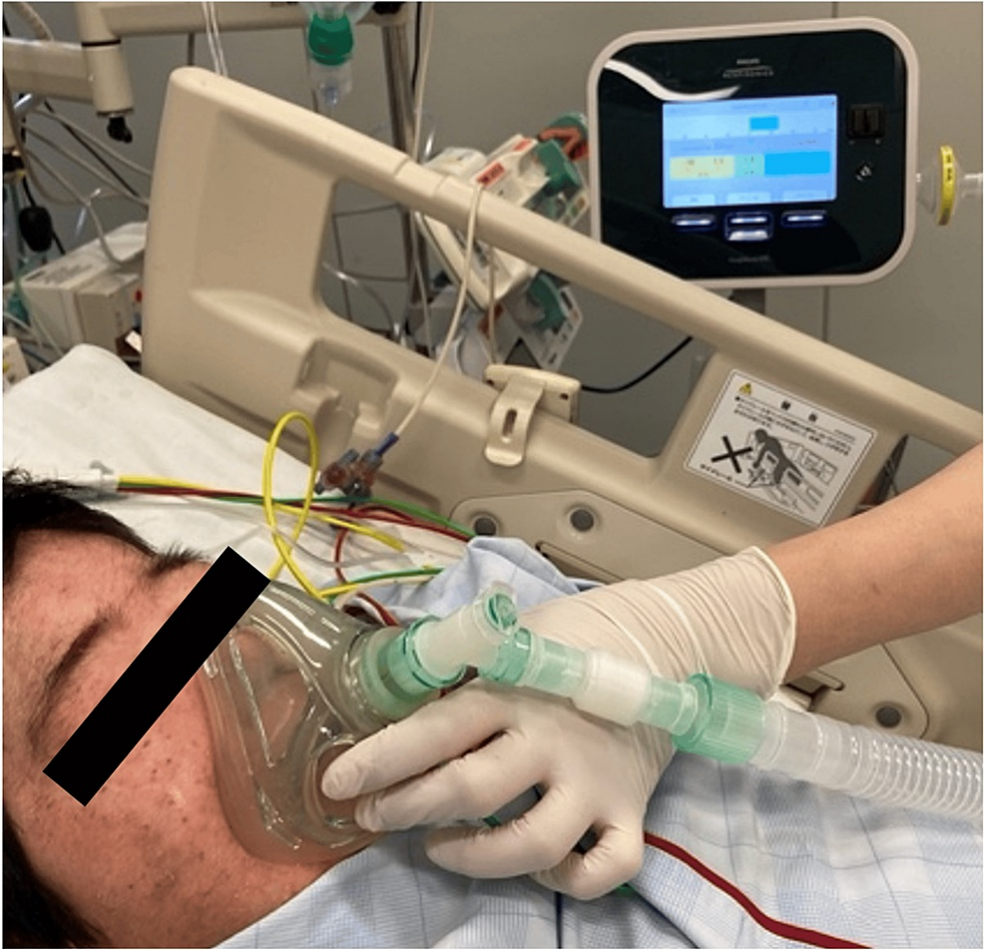
Overview of the use of MI-E on a current patient The MI-E is a compact and simple medical device that can be conveniently used at the bedside. Physicians and nurses assisted with mask fitting. MI-E: mechanical insufflation-exsufflation

A 32-year-old woman on long-term steroid therapy for Blau syndrome (height, 100 cm; weight, 29 kg; body mass index, 29 kg/m^2^) presented to our emergency department with dyspnea. Her mother had previously had a positive severe acute respiratory syndrome coronavirus2 (SARS-CoV-2) polymerase chain reaction (PCR) test. Based on a SARS-CoV-2 PCR test and lung computed tomography, our patient was diagnosed with acute respiratory distress syndrome due to coronavirus disease 2019 pneumonia. Upon admission, her vital signs were as follows: heart rate, 118 beats/min; blood pressure, 150/75 mmHg; respiratory rate, 28 breaths/min; percutaneous arterial oxygen saturation (SpO_2_) on oxygen inhalation (6L/min), 95%; and body temperature, 36.8℃. High-flow nasal cannula oxygen therapy (HFNC) with an initial setting of 60% oxygen concentration and a flow rate of 25 L/min was initially applied to improve oxygenation. However, hypoxemia persisted and intubation was performed on the same day. Given the refractory hypoxemia, veno-venous extracorporeal membrane oxygenation (VV-ECMO) was established on the following day. Subsequently, her lung condition improved (Figure 1A), and she was weaned from VV-ECMO on the 10th day and extubated on the 13th day. HFNC with a setting of 30% oxygen concentration and a flow rate of 25 L/min was applied for post-extubation respiratory assistance. After a few hours, she gradually developed tachypnea and unstable SpO_2_. Chest radiography performed on the 14th day revealed massive atelectasis in the left lung (Figure 1B). Expectorant use and manual respiratory physiotherapy did not improve the sputum amount suctioned through the oral cavity. Reintubation was considered due to deterioration of hypoxemia and tachypnea (SpO_2_ on 60% inspired oxygen, 93%; respiratory rate, 55 breaths/min). To facilitate mucus clearance, we initiated MI-E (Cough Assist E70, Philips Respironics, Murrysville, PA, USA) with inspiratory and expiratory pressures of +30 cmH_2_O and -30 cmH_2_O, respectively, for 1.5 seconds (Figure 2). It increased sputum expectoration and improved her respiratory status. Chest radiography performed shortly after MI-E initiation revealed a dramatic decrease in atelectasis (Figure 1C). MI-E was continued for two days and HFNC was discontinued on the 16th day. The patient was discharged from the ICU on the 21st day, without any recurrence of respiratory deterioration.

## Discussion

Our findings indicate that airway secretion management using MI-E can effectively prevent reintubation in patients with post-extubation acute respiratory failure.

Even among patients who meet all criteria for weaning, 10%-20% of patients present extubation failure [6], which is mainly caused by airway disturbance [1] partly attributable to secretion encumbrance. Bronchodilators, expectorants, postural drainage, and airway suctioning are often used for standard airway secretion management in the ICU; however, they cannot effectively stimulate natural coughing.

MI-E is effective as a supportive tool for cough augmentation in patients with NMD. The American Association for Respiratory Care guidelines [2] recommend the use of MI-E in adult and pediatric patients with NMD, respiratory muscle weakness, or cough disorders, especially in cases where the peak cough flow is <270 L/min. de Camillis et al. [7] reported that in ICU patients receiving mechanical ventilation for > 24 h, respiratory physiotherapy with the MI-E device yielded a significantly higher weight of aspirated airway mucus and static lung compliance than standard respiratory physiotherapy alone. Further, there were no significant between-intervention differences in airway resistance, breathing work, and complication rates. Gonçalves et al. [8] showed that the use of MI-E thrice a day in patients mechanically ventilated for >48 h reduced the reintubation rate. Most of the studies were performed on adults without hereditary diseases. Contrastingly, we observed the efficacy of MI-E in a patient with Blau syndrome, which is a congenital anomaly of the respiratory muscles and skeletal system.

We applied MI-E on the patient after we detected the post-extubation respiratory distress. In some previous reports, MI-E has been used before or immediately after extubation, and there have been reports of stable respiratory status after extubation. In the future, it may be necessary to discuss when to start using MI-E in patients with impaired coughing or expectorant function. A randomized controlled trial [9] is currently underway to evaluate the use of MI-E in acutely ventilated patients before extubation.

The widespread application of MI-E is impeded by a lack of expert knowledge and experience, especially for critically ill patients on ventilators [10,11]. This report may contribute toward the elucidation and promotion of MI-E use in this field.

## Conclusions

Our findings demonstrate the efficacy of MI-E in treating acute cough impairment after extubation. Although the widespread use of MI-E on acutely ill patients is still hampered by a lack of expert knowledge and experience, this report suggests that MI-E has great potential to be an efficient airway clearance technique in the ICU. We expect our findings to contribute to further understanding and prevalence of MI-E use in critical care settings.
